# Sustainable eye care at Kitale Eye Unit

**Published:** 2013

**Authors:** Hillary Rono

**Affiliations:** Ophthalmologist: Kitale District Hospital, Kitale, Kenya. **hkrono@yahoo.com**

**Figure:**
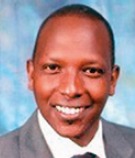
Hillary Rono

Eye care services are an integral part of health services. It is therefore important that eye care programmes are designed with financial sustainability in mind so they can become self-sufficient within a reasonable period of time. This article describes how costs were kept low, income was generated, and quality eye care was maintained at Kitale Eye Unit, a department within the Kitale District Hospital, the county referral hospital for Transzoia (population 1 million) in Kenya.

The unit has one ophthalmologist, three ophthalmic clinical officers and six nurses. They are all employed by the ministry of health. Four support staff (clerks, cleaners and cashiers) are employed by the hospital.

Capital development costs, such as building and equipping the eye unit, start-up costs of training personnel and support for outreach programmes were provided by Operation Eyesight Universal (OEU).

About 60% of the recurrent expenditure is funded by government, 18% comes from internally generated revenues, and the rest (22%) is supported by partner non-governmental organisations (NGOs).

## Keeping costs low

### Human resources management

The people are the most important asset in any organisation. Eye care workers are scarce and expensive to recruit. Various methods have been used to utilise available staff optimally, for example training, motivation, target setting, and task shifting. These will be discussed in more detail in a future issue of the *Community Eye Health Journal*.

**Figure:**
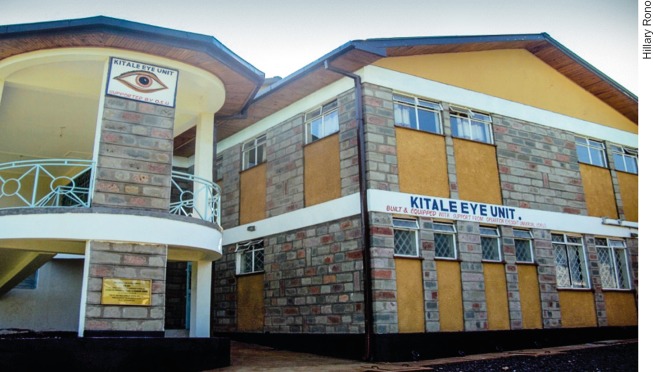
Kitale eye unit was built and equipped with support from Operation Eyesight Universal.

### Time management

There are three surgical days in a week. At first, operations are booked on just the first day. Once this day is full, bookings will be made on the next surgical day and so on. This maximises use of theatre space as well as the surgeon's time.

### Supplies: surgical consumables

One of our goals is to make eye care affordable by reducing the cost of surgical consumables. This has been achieved as follows.

1. Consumables for cataract surgery are purchased in bulk directly from manufacturers, reducing the costs by up to 50%.
2. A trachoma surgery kit with essential consumables has been developed in collaboration with Deepak Enterprises in India. This has made it cheaper to conduct trachoma surgery.
3. All surgeons perform small-incision sutureless cataract surgery as day surgery. Admissions are at the patient's request or when there are complications that necessitate it. The costs associated with overnight admission and sutures are therefore avoided.

## Increasing income

We have several ways of increasing income in Kitale.

### Fee for service (user fees)

All patients pay for the services provided. This fee can be paid by their insurance or out of pocket for those without medical insurance. The consultation fee and user fee for procedures are levied as per hospital guidelines. The funds generated go to a common hospital pool and quarterly budgets are developed to spend the revenue.

### Ophthalmic pharmacy

An ophthalmic products pharmacy was established with seed support from OEU. Medicine was procured in bulk, directly from the manufacturers.

A profit margin of 30% was added to the cost of eye drops. This profit is divided as follows:

- one third to support the outreach programme
- one third to the hospital to pay utility bills
- one third to increase stocks.

In 2011 and 2012, the proceeds from the pharmacy supported two surgical camps and the surplus was enough to sponsor 70 cataract operations.

### Outreach

Eye drops and spectacles for reading are sold during outreach services to those who can afford to pay. The proceeds are used to provide transport and meals for eye workers.

**Figure:**
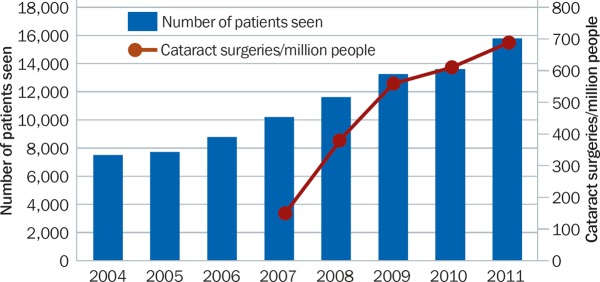
Figure 1. Patients seen and cataract surgical rate at Kitale eye unit

### Local philanthropists

We have partnered with local service clubs, companies and philanthropists to support patients who are poor. The clubs include Lions clubs, which support surgical camps and trachoma activities.

## Maintaining quality

Maintenance of the quality of eye services is continuous and dynamic. The three key processes are:

1. Identification of areas that need improvement.
2. Analysing areas of difficulty and proposing solutions.
3. Monitoring progress and providing feedback to staff.

At Kitale, identification of areas that need improvement is achieved through auditing surgical outcomes (such as cataract outcome) and getting feedback from patients about their experiences at the eye unit. A staff meeting is held every 2–3 months to discuss surgical outcomes and patient feedback. Probable solutions are also identified. Some of the solutions proposed so far have included purchase of essential equipment, continuous medical education for staff to keep them updated about current management of eye conditions, customer relations training, and refresher training for cataract surgeons.

Monitoring of progress and feedback to staff is done through quarterly reports, which are prepared by the eye department. Supportive supervision by Kenya's national eye coordinator motivates staff to achieve better quality. Output has increased as a result (Figure 1).

## Conclusion

In every unit, the eye care team is responsible for diversifying and improving quality of services in order to attract all clients, both those without funds and those who can pay for service. At the same time, managers have to be innovative in staff motivation, revenue generation, community support, political support, and hospital administration support. These days, government policies in Kenya allow innovation and freedom to think outside the box.

*The author would like to acknowledge Michael Gichangi, Chief Ophthalmologist, Ministry of Health, Kenya for his contribution*.
